# Workshop report—Vulnerability in multi-hazard risks: Addressing its complexity and dynamics

**DOI:** 10.1016/j.isci.2026.115250

**Published:** 2026-03-18

**Authors:** Alexandre Pereira Santos, Silvia De Angeli, Franziska Stefanie Hanf, Mirbach Charlotta, van Maanen Nicole, Vitus Benson, de Ruiter Marleen Carolijn, Alexandre Dunant, Stefano Terzi, Pia-Johanna Schweizer, Taís Maria Nunes Carvalho, Mariana Madruga de Brito, Kelley De Polt, Robert Šakić Trogrlić, Marc van den Homberg

**Affiliations:** 1 Department of Geography, Ludwig-Maximilians-Universität München, Munich, Germany; 2 Université de Lorraine, CNRS, LIEC, Metz, France; 3 Atmospheric Science, Department of Earth System Sciences, Faculty of Mathematics, Informatics and Natural Sciences, Earth and Society Research Hub (ESRAH), University of Hamburg, Hamburg, Germany; 4 Research Institute for Sustainability at GFZ Helmholtz Centre for Geosciences, Potsdam, Germany; 5 Institute for Environmental Studies, Vrije Universiteit Amsterdam, Amsterdam, the Netherlands; 6 Department Biogeochemical Integration, Max Planck Institute for Biogeochemistry, Jena, Germany; 7 Center for Climate Change and Transformation, Eurac Research, Bolzano, Italy; 8 Helmholtz Centre for Environmental Research, Leipzig, Germany; 9 Advancing Systems Analysis Program, International Institute for Applied System Analysis, Laxenburg, Austria; 10 The Netherlands Red Cros’ Data and Digital Team 510, The Hague, the Netherlands; 11 Faculty of Geo-Information Science and Earth Observation, University of Twente, Enschede, the Netherlands

## Abstract

In November 2025, an interdisciplinary group of vulnerability researchers met in Munich and identified three challenge-opportunity clusters: first, overcoming epistemological divides to enable meaningful interdisciplinary integration. Second, the interoperability of data, methods, and evidence can strengthen robustness and policy relevance. Third, vulnerability assessments must adopt fit-for-purpose levels of complexity that preserve local context while enabling cross-scalar translation. This backstory is a call-to-action to accelerate the transition of the field toward robust, policy-salient, and socially legitimate integrative and interdisciplinary research.

**Figure:**
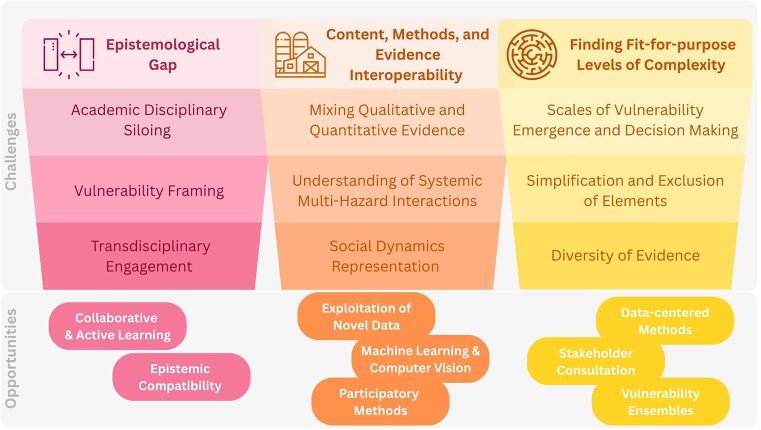
Above image: Framing the three challenge-opportunity clusters. Source: image by K. De Polt, with icons by Flaticon.com.

The vulnerability research field matures and faces challenges in epistemological integration, data and methods interoperability, and managing the complexity of socio-environmental dynamics across multiple scales.

By integrating data-centric methods with citizen participation, local and non-academic knowledge, research can identify blind spots, enhance the legitimacy of evidence, and identify long-standing root causes of social vulnerability drivers. It is imperative that the selection of represented groups is careful and transparently acknowledges those missing to foreground the role of culture, law, social organization, and power in shaping vulnerability.

## Main text

### Beginnings

Given the rising frequency and severity of climate hazards, it becomes ever more urgent to address the complexities of vulnerability while accounting for its dynamics.^1^ Indeed, while the impacts of hazards themselves cannot be mitigated entirely, we can reduce the vulnerability of the elements at risk.^2^ This is a complex task given the systemic nature of global society, the increasing frequency and intensity of multiple hazards, and the context-specific vulnerabilities across regions and communities.^3^ Existing efforts have prioritized either depth (through integration) or breadth (through diversity and coverage) in understanding vulnerability dynamics and their complexity.^4^ Different scientific communities seek to tackle this challenge, often exploring emerging opportunities in interoperability, data sharing, and interdisciplinary research.^5^ However, effective integration is still missing, hindering effective policy and decision-making.

A workshop in Munich in November 2025 brought together a diverse range of scholars to promote a deeper interdisciplinary integration and accelerate an interdisciplinary transition perceived in the field. Scholars from sociology, geography, environmental engineering, earth sciences, sustainable climate change adaptation, and disaster risk management came together to chart key challenges for deeper integration and to lay the groundwork for new, better-delimited research avenues. This meeting was jointly organized by LMU, Universität Hamburg, Université de Lorraine, Vrije Universiteit Amsterdam, and supported by the LMUExcellent Postdoc Support Fund (PSF), a central funding tool of Ludwig-Maximilians-Universität (LMU), Munich, Germany. In the next sections, we present the motivation and results of this workshop, which also demonstrates the power of early-career research funding to foster scientific innovation.

### Motivation

The initial motivation to explore this topic came from a splinter meeting held during the European Geosciences Union 2025 General Assembly in Vienna. There, researchers mapped three key challenges to addressing vulnerability: (i) accounting for the temporal and spatial dynamics of vulnerability, (ii) exploring new methods and data, and (ii) overcoming the systemic, cross-sectoral, and disciplinary boundaries in the field. Previous research also revealed that these challenges could become opportunities and entry points into the complexities of vulnerability. For example, studies have emphasized the need to articulate information across social sectors, spatial and temporal scales, and hazard types^4^ and to integrate stakeholders, citizens, and local knowledge in the research process. Work on systemic risk cycles has revealed the complex interconnections between multiple hazards, socioeconomic dynamics, governance, and climate adaptation, highlighting how self-reinforcing barriers can hinder adaptation and even increase risk.^6^ Research on multi-hazard risk management emphasized the need for new approaches to integrate vulnerability that can account for temporal dynamics and the effects of recovery processes in shaping new vulnerability conditions.^7^ Methodologically, prior research identified potential in novel Earth Observation (EO) methods and data to assess vulnerability across geographies.^8^ Additional capacity was found in interoperating data across domains (i.e., multi-modal data analysis), with the support of machine learning approaches.^5^

Based on this initial mapping of challenges and the potential innovations, we reconvened in Munich. The event included a pedagogical exercise to foster interdisciplinary understanding and two sessions for in-depth discussion. This event resulted in three challenge-opportunity clusters presented in Image 1 to address the dynamics and complexities of vulnerability, to which this report turns next.

### Challenges and opportunities for addressing dynamic vulnerability across multiple hazards

The vulnerability research field matures and faces challenges in epistemological integration, data and methods interoperability, and managing the complexity of socio-environmental dynamics across multiple scales. In this section, we reframe these challenges as opportunities to propel innovation and strengthen policy relevance in the field.

#### Cluster 1: The epistemological gap

Vulnerability research has been interdisciplinary since its inception,^9^ and recent advances in the field point to the convergence of multiple research streams (e.g., coupled hazards, multiple stressors, and systemic risks).^4^ Even so, a key obstacle for integration is the persistence of disciplinary siloing within academic practice that continues to impede meaningful integration. This gap is not merely organizational or taxonomic, but reflects deeper differences in how vulnerability is conceptualized, operationalized, and normatively framed across disciplines. These differences are evident, for example, in the co-existence of multiple, only partially compatible definitions of vulnerability within disaster risk reduction and climate risk research. Differences in terminology often signal more fundamental disagreements concerning causality, scale, temporality, and the role of agency and structure. As a result, concepts require constant (re-)definition, findings from adjacent disciplines are treated with epistemic caution, and intertheoretical collaboration is frequently reduced to parallel rather than integrative work. Importantly, this fragmentation also constrains transdisciplinary engagement, limiting the meaningful inclusion of non-academic knowledge despite widespread recognition of its relevance for understanding context-specific vulnerability.

Addressing these issues requires not only creating opportunities for collaboration but also active learning experiences that help researchers recognise and move beyond their own assumptions, values, and limitations embedded in their own disciplinary positions. During the workshop, we introduced an ice-breaking exercise that asked the participants to identify which researcher among them had proposed each of the anonymous individual research challenges displayed. This activity proved effective in helping researchers approach vulnerability from new perspectives, as frequent misattributions revealed both unexpected convergences and unacknowledged distances among participants, prompting reflection on how disciplinary identities shape problem framings. Hence, we understand that future research should implement pedagogical devices and practices that mediate disciplinary positioning. Furthermore, involving local and non-academic knowledge can reveal context-specific drivers of vulnerability that are often invisible otherwise.

Notwithstanding these challenges, there have already been successful interdisciplinary and intertheoretical efforts.^4^^,^^5^ Scientific pragmatism has encouraged an emphasis on usefulness and problem-solving across disciplinary boundaries, establishing intertheoretical connections that allow for increased testing, validation, and calibration of vulnerability assessments.^10^ Conceptual advances in tackling complexity^3^ in using mixed methods to translate evidence,^4^ and integrative theory-building^11^ point to increased articulation, rather than replace, disciplinary perspectives. Cross-disciplinary collaboration allows advances in adjacent communities to help overcome barriers and mitigate the risk of research “spinning in circles”^11^ due to disciplinary isolation. However, without explicit attention to epistemic compatibility and power relations in knowledge production, such efforts risk producing assemblages of concepts rather than coherent frameworks.

#### Cluster 2: Content, methods, and evidence interoperability

Building on the first challenge-opportunity pair, the participants focused on research implementation. They highlighted a need to overcome the divergence between quantitative and qualitative research methods, which hinders nuanced representation of vulnerability in global datasets, for example. Additionally, there are no common vulnerability conceptual frameworks to foster collaboration and comparison of data or evidence. Additionally, there is a lack of understanding of hazard interaction, both with other hazards and across social sectors, institutions, and governance processes.^12^ There remain large gaps in coverage at the subnational scale, especially at neighborhood and finer scales that restrict analyses at these levels, despite advances in data acquisition and management. Overall, this divide reflects not only methodological preferences but also different assumptions about what constitutes valid evidence, how uncertainty should be handled, and which aspects of vulnerability are deemed measurable or policy relevant.

In response, the participants found opportunities to operationalize integrated research. First, research may combine data on multiple hazards to enable forensic analysis of their impacts and the vulnerability conditions of the affected populations.^10^ Systematic collection and curation of vulnerability data and case-study evidence could further support comparative and replication studies, strengthening robustness and cumulative learning. Another avenue includes leveraging machine learning and computer-vision methods to detect, track, and eventually anticipate patterns across space and time. While empirical data on vulnerability is often missing, a great deal of it is hidden in unstructured data (e.g., archival records, text, or images lacking indexing). EO is also promising in providing data at increased resolutions and multiple domains (population, the built and natural environments, or climate).^8^ However, these approaches also risk privileging what is observable and quantifiable, often misrepresenting social dynamics, power relations, and human behavior. Of note is the potential to establish shared standards on data and methods that can bridge the social, environmental, and hazard dimensions of vulnerability.^5^ Yet, these standards should not be understood as neutral, but as negotiated epistemic infrastructures that shape evidence framing. In this context, integrating participatory methods (e.g., real-world labs) in vulnerability assessments has contributed to revealing local, context-dependent drivers, increasing assessment and policy legitimacy. By integrating data-centric methods with citizen participation, local and non-academic knowledge, research can identify blind spots, enhance the legitimacy of evidence, and identify long-standing root causes of social vulnerability drivers.^12^ It is imperative that the selection of represented groups is careful and transparently acknowledges those missing to foreground the role of culture, law, social organization, and power in shaping vulnerability.^13^

#### Cluster 3: Finding fit-for-purpose levels of complexity

Another challenge lies in how vulnerability research tackles complexity. While past research has often resorted to simplification (i.e., eliminating factors, drivers, actors, or geographies that fall outside the analytical scope), there is growing criticism of how simplified vulnerability depictions reinforce inequalities. This is manifest in the mismatch between where vulnerability is produced and manifested, and the decision-making aimed at addressing it. On the one hand, vulnerability manifests primarily at the local scale, shaped by household, neighborhood, or community-level conditions, even if informed by broader dynamics (e.g., colonial legacies). On the other hand, decisions on vulnerability are often taken at broader scales (e.g., national or regional), ignoring context-specific nuance and reinforcing inequalities that disproportionately burden those most vulnerable.^13^ Conversely, studies seeking holistic approaches are often overburdened by evidence diversity, competing causal explanations, or complicated system representations that lack empirical grounding and analytical capacity.

While a universal conceptual and methodological vulnerability framework is neither desirable nor feasible, vulnerability research would greatly benefit from flexible, fit-for-purpose best practices. The participants identified that the level of complexity of vulnerability assessments should fit their purpose, including different spatial scales (e.g., national, regional, or local), temporal coverage (historical or future-oriented, short or long-term), or hazard context (e.g., single or multi-hazard). Explicitly aligning concepts and methods with these purposes may support more transparent cross-scalar translation and comparative or transferable vulnerability definitions without erasing contextual specificity.

To enable this calibration, research may build upon the systematically collected case study evidence mentioned above and implement flexible methods (e.g., boundary objects and methods)^14^ that tolerate ambiguity and support coordination between epistemic communities without enforcing false precision. Machine learning methods offer similar potential to detect and track patterns across space and time^15^ but demand adequate theoretical framing to clarify their legitimacy. Data on multiple hazards may further leverage data-centred methods, especially when combined with strong stakeholder consultation across a set of geographies. Such ensembles may allow advancing the interoperability of concepts, methods, and data across socio-environmental systems and institutions.^5^ Such an approach to complexity, when guided by purpose rather than ambition, offers a pathway toward more integrative and equitable vulnerability research, with tangible implications for both science and policy.

### Where do we go from here?

Vulnerability research is undergoing a significant transition. As the field matures, it increasingly confronts limits in epistemological integration, data and methods interoperability, and the management of complexity. Reframing these challenges as opportunities can accelerate innovation and strengthen policy relevance. The key points of this transition are.

1. Effective vulnerability research requires explicit integration across disciplines, methods, and scales. Fragmentation in concepts, evidence, and assumptions continues to limit policy relevance and comparability.
2. Interoperability is essential for actionable vulnerability assessments. Shared, transparent approaches to data, methods, and standards can improve robustness while shaping which vulnerabilities become visible in decision-making.
3. Local context must be preserved when scaling vulnerability information. Loss of contextual detail during upscaling risks reinforcing inequalities and misdirecting interventions, resulting in maladaptation.
4. Vulnerability assessments should be fit-for-purpose rather than universally complex. Aligning analytical complexity with policy goals, spatial scale, and hazard context improves usability and equity.
5. Combining data-driven and participatory approaches strengthens legitimacy and insight. Integrating computational methods with stakeholder engagement reveals blind spots and structural drivers of vulnerability.
6. Early-career and interdisciplinary funding play a catalytic role. Targeted support enables experimentation, epistemic learning, and innovation critical for advancing integrative vulnerability research.

By bringing together a diverse group of scholars in a collaborative yet disciplinary-questioning environment, the workshop on vulnerability dynamics foregrounded a set of opportunities for the field. This backstory should be seen as a call-to-action for researchers and practitioners aligned with this vision or who seek to contribute to the dialogue on the transition of vulnerability research currently apace.

## Acknowledgments

The authors would like to thank the promoting institutions, namely Ludwig-Maximilians-Universität München, Universität Hamburg, Université de Lorraine, and Vrije Universiteit Amsterdam. The LMUExcellent Postdoc Support Fund (PSF), a central funding tool of Ludwig-Maximilians-Universität (LMU), Munich, Germany, provided the funding for this workshop.
