# Workshop report—Vulnerability in multi-hazard risks: Addressing its complexity and dynamics

**DOI:** 10.1016/j.isci.2026.115452

**Published:** 2026-04-02

**Authors:** Alexandre Pereira Santos, Silvia De Angeli, Franziska Stefanie Hanf, Charlotta Mirbach, Nicole van Maanen, Vitus Benson, Marleen Carolijn de Ruiter, Alexandre Dunant, Stefano Terzi, Pia-Johanna Schweizer, Taís Maria Nunes Carvalho, Mariana Madruga de Brito, Kelley De Polt, Robert Šakić Trogrlić, Marc van den Homberg

## Main text

(iScience *29*, 115250; April 17, 2026)

In the originally published version of this Backstory, due to typesetter error, three author names were incorrectly formatted, with the last names appearing before the first names. The name “Mirbach Charlotta” should properly refer to the author “Charlotta Mirbach,” the name “van Maanen Nicole” should properly refer to the author “Nicole van Maanen,” and the name “de Ruiter Marleen Carolijn” should properly refer to the author “Marleen Carolijn de Ruiter.” These author names have been corrected online, and Production apologizes for these errors.

